# Supplementary material for: Utilization of a Histoplasma capsulatum zinc reporter reveals the complexities of fungal sensing of metal deprivation
Source: mSphere. 2024 Jan 23;9(2):e00704-23. doi: 10.1128/msphere.00704-23 (PMC10900905; doi:10.1128/msphere.00704-23)
Supplement: Supplemental Figures — Figures S1 to S4. [file msphere.00704-23-s0001.pdf]

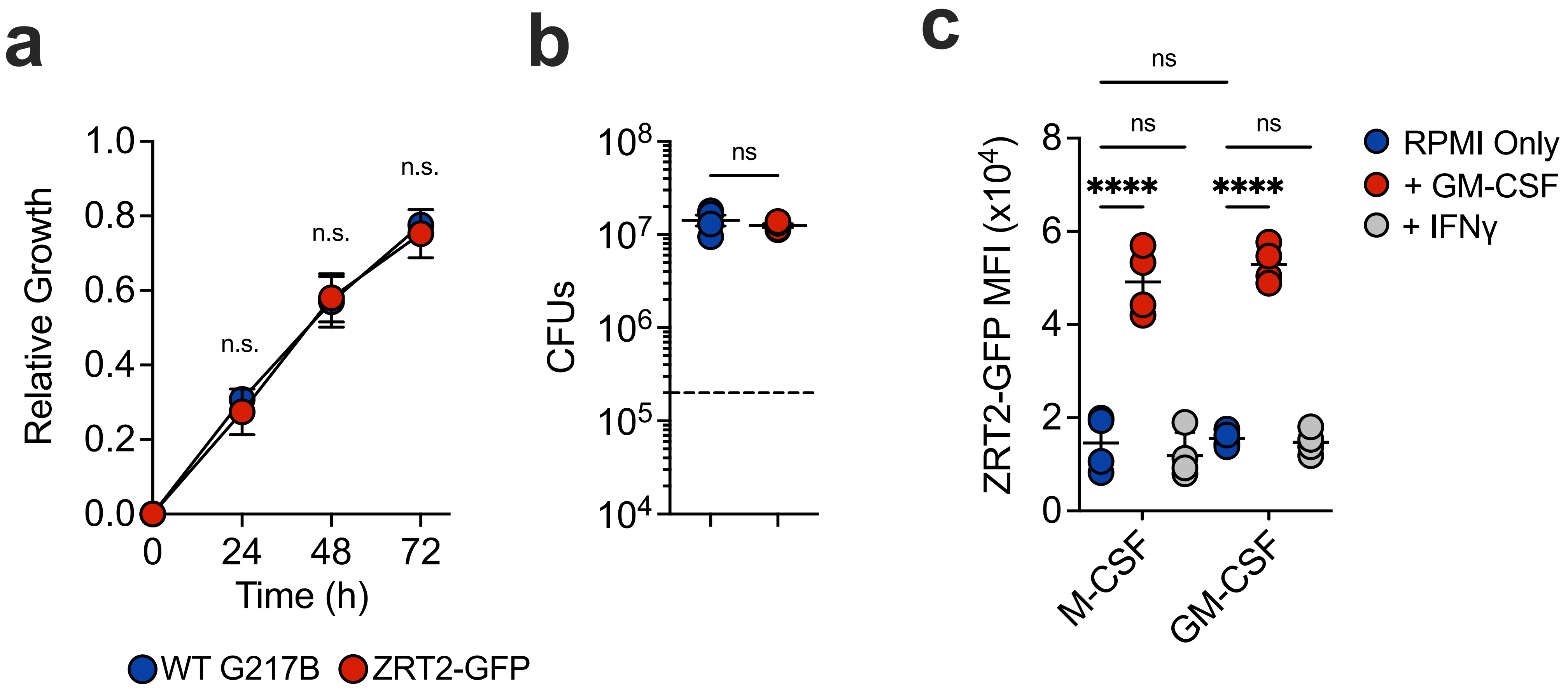
a
Relative Growth
1.0
0.8
0.6
0.4
0.2
0.0
n.s.
n.s.
n.s.
0
24
48
72
Time (h)
WT G217B
ZRT2-GFP
b
CFUs
ns
c
ns
ns
ns
****
****
ZRT2-GFP MFI (x10^4)
8
6
4
2
0
M-CSF
GM-CSF
RPMI Only
+ GM-CSF
+ IFNγ

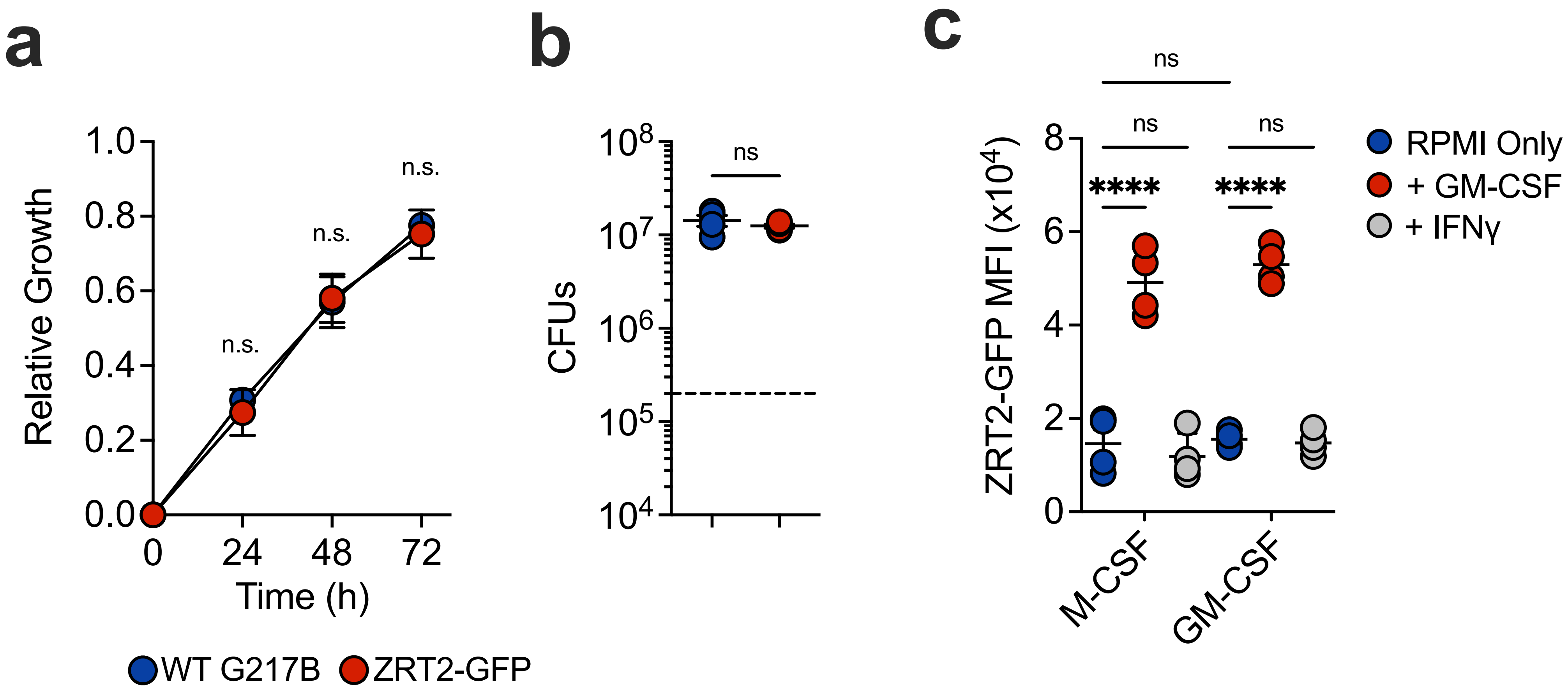

a
b
c
Relative Growth
1.0
0.8
0.6
0.4
0.2
0.0
0
24
48
72
Time (h)
n.s.
n.s.
n.s.
WT G217B
ZRT2-GFP
CFUs
10^8
10^7
10^6
10^5
10^4
ns
ZRT2-GFP MFI (x10^4)
8
6
4
2
0
ns
ns
ns
****
****
M-CSF
GM-CSF
RPMI Only
+ GM-CSF
+ IFNγ

Figure S1. The Zn reporter strain exhibits no defects in growth or virulence. (**a**) Growth of WT and ZRT2-GFP *H. capsulatum* yeasts in liquid HMM. (**b**) CFUs or ZRT2-GFP *H. capsulatum* yeasts recovered from mouse lungs 7-dpi. (**c**) ZRT-GFP expression in response to GM-CSF by BMDM differentiated in the presence of M-CSF or GM-CSF.

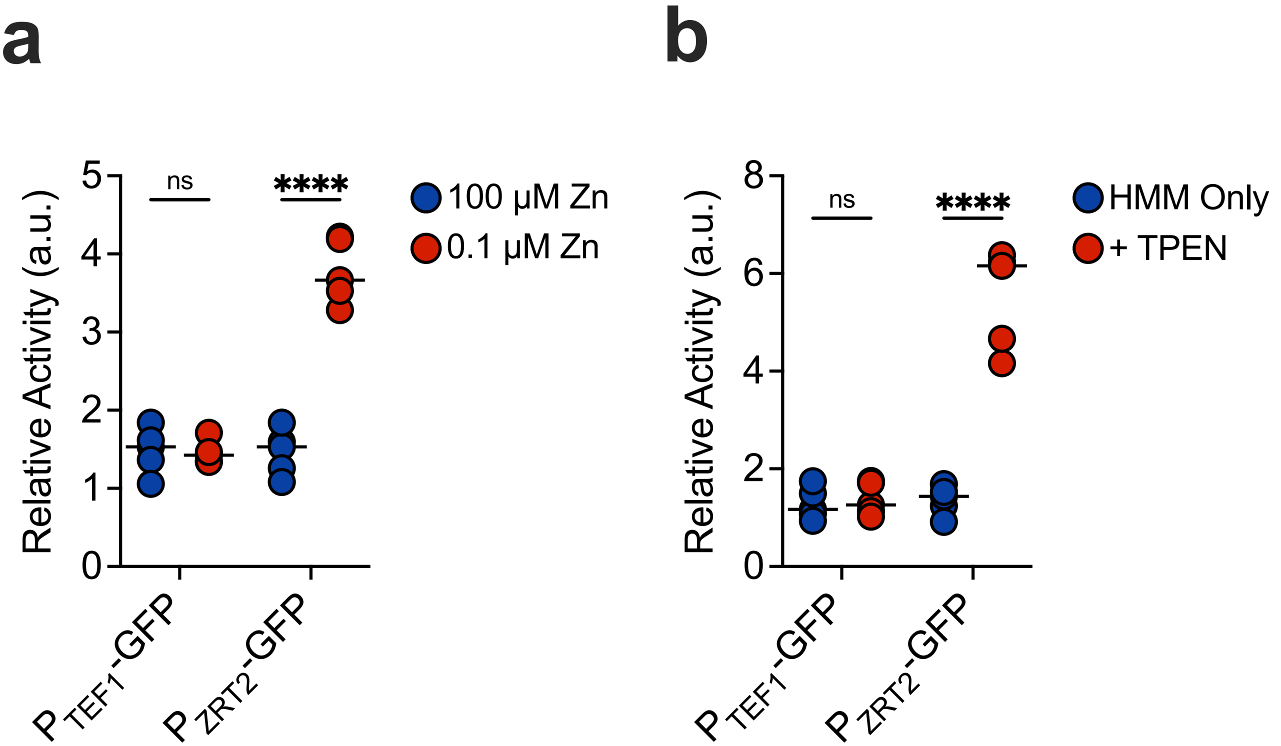

a
Relative Activity (a.u.)
5
4
3
2
1
0
ns
****
100 µM Zn
0.1 µM Zn
P TEF1-GFP
P ZRT2-GFP
b
Relative Activity (a.u.)
8
6
4
2
0
ns
****
HMM Only
+ TPEN
P TEF1-GFP
P ZRT2-GFP

Figure S2. The Zn reporter strain responds to Zn-limited growth conditions. (**a**) Expression of ZRT2-GFP or TEF1-GFP *H. capsulatum* yeasts in chelated liquid HMM supplemented with 100 µM $ZnSO_4$ or 0.1 $ZnSO_4$, respectively. (**b**) Relative activity of ZRT2-GFP or TEF1-GFP *H. capsulatum* yeasts in liquid HMM treated with 10 µM TPEN or vehicle. dTomato and GFP fluorescence was measured 48 hours after the start of experiments. **** = $P < 0.0001$

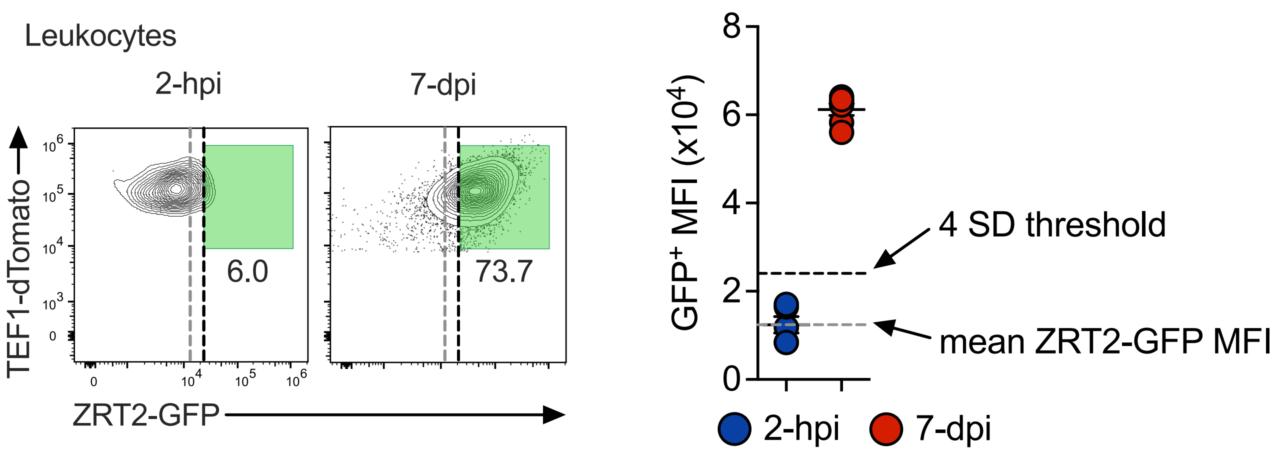

Leukocytes
2-hpi
7-dpi
TEF1-dTomato
ZRT2-GFP
6.0
73.7
0
10^4
10^5
10^6
10^3
GFP+ MFI (x10^4)
8
6
4
2
0
4 SD threshold
mean ZRT2-GFP MFI
2-hpi
7-dpi

Figure S3. Setting ZRT2-GFP threshold for in vivo measurement of reporter activity. WT mice were infected with 2 x 10^5 ZRT2-GFP *H. capsulatum* yeasts i.n. and sacrificed at 2-hours post-infection (2-hpi) and 7-days post-infection (7-dpi) and ZRT2-GFP MFI was calculated. 4 standard deviations (SD) from the mean MFI at 2-hpi was determined as the threshold for the ZRT2-GFP reporter to be "on" vs. "off."

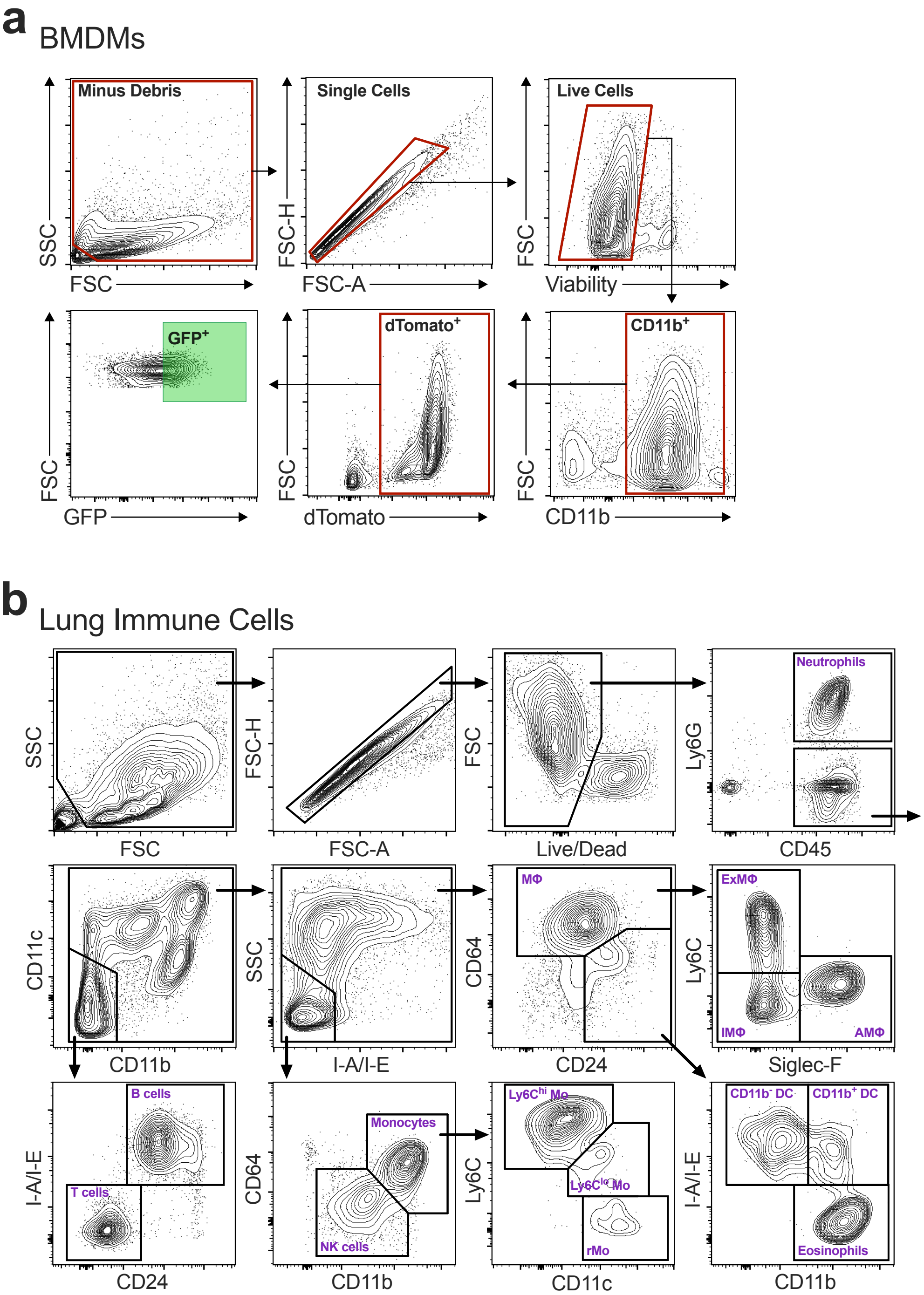
a
BMDMs
Minus Debris
SSC
FSC
Single Cells
FSC-H
FSC-A
Live Cells
FSC
Viability
CD11b+
FSC
CD11b
dTomato+
FSC
dTomato
GFP+
FSC
GFP
b
Lung Immune Cells
SSC
FSC
FSC-H
FSC-A
FSC
Live/Dead
Neutrophils
Ly6G
CD45
CD11c
CD11b
SSC
I-A/I-E
MΦ
CD64
CD24
ExMΦ
IMΦ
AMΦ
Ly6C
Siglec-F
B cells
T cells
I-A/I-E
CD24
Monocytes
NK cells
CD64
CD11b
Ly6Chi Mo
Ly6Clo Mo
rMo
Ly6C
CD11c
CD11b- DC
CD11b+ DC
Eosinophils
I-A/I-E
CD11b

Figure S4. Spectral Flow Cytometry Gating Strategies. Gating strategies for (**a**) BMDMs and (**b**) lung immune cell populations. Data was acquired on a Cytek Aurora Spectral Cytometer.
