# Supplementary material for: Utilization of a Histoplasma capsulatum zinc reporter reveals the complexities of fungal sensing of metal deprivation
Source: mSphere. 2024 Jan 23;9(2):e00704-23. doi: 10.1128/msphere.00704-23 (PMC10900905; doi:10.1128/msphere.00704-23)
Supplement: Supplemental Tables — Tables S1 to S4. [file msphere.00704-23-s0002.pdf]

Table S1: *H. capsulatum* Strains

strain	Genotype[1]
G217B	Wild type NAm2 isolate (ATCC 26032)
WU15	*ura5-42Δ*
OSU233[2]	*ura5-42Δ zzz:pQS01 (apt3,* P_{TEF1}*-rfp)*
OSU326[2]	*ura5-42Δ zzz:pCR623 (URA5,* P_{TEF1}*-gfp)*
UC25	*ura5-42Δ zzz:pQS01 (apt3,* P_{TEF1}*-rfp) zzz:pLB111 (URA5,* P_{ZRT2}*-gfp)*

[1] gene designations:

zzz:: plasmid integration at an undetermined chromosomal location

apt3: aminoglycoside phosphotransferase (G418 resistance)

gfp: green-fluorescence protein (E*GFP*)

rfp: red-fluorescence protein (tdTomato)

URA5: orotate phosphoribosyltransferase

TEF1: translation elongation factor EF-1α

ZRT2: high affinity zinc transporter

[2]source: Shen Q, et al., 2018 reference 18

Table S2: PCR Primers Used in This Study

Primer Number	Name	Sequence
P1	ZRT2-AvrII	CGGTCCTAGGCGGGGTCCATGCTGGGGATTTAGTTTG
P2	ZRT2-AscI	TAGGCGCGCCATGGCTGGCGAAACGAAG
P3	pCR623-GFP-5'	CCATGTGATCGCGCTTCT
P4	pZRT2-5'	GCGGTGCTGAGTCAGTTATT
P5	pTEF1-5'	TAGCATTGTAACATCTGGGACTG

Table S3: qPCR Primers Used in This Study

Gene	Sequence Direction[1] (5' to 3')	
	Forward	Reverse
GAPDH	TATTGGGCGTATTGTCTTC	GGTCTCTCTCTTGGTAGAA
ZRT1	TCACCTTCCACCAAACCTTC	CATTCGTCATCCCTACCATCAG
ZRT2	TCCTCGAATTCGGCATCATT	ATGGAAATTGGAGTAGAGAGGC
ZRT3	GTGTCAGTGGCATTGCTTG	GCTAGAAGAGAGTCCAGCTTTA
ZAP1	CCTGACACATAGTAACCAACAAAGG	CGCCAGTATGAATTCGCATGT
ZRC1	GAACTAGTCGTTGGTTATT	AGATGGAAAGACATAATGC

[1] Direction relative to mRNA transcript

Table S4: Flow cytometry antibodies

Marker	Fluorophore	Clone	Company	Cat. No.
Siglec-F	BV421	S17007L	Biolegend	155509
Ly6C	BV510	HK1.4	Biolegend	128033
CD64	BV605	X54.5/7.1	Biolegend	139323
MHCII	BV711	M5/114.15.2	Biolegend	107643
Ly6G	AF-700	1A8	Biolegend	127622
CD24	Pacific Blue	M1/69	Biolegend	101820
F4/80	PE-Cy5	BM8	Biolegend	123112
CD11c	PE-DZ594	N418	Biolegend	117347
CD11b	APC-Cy7	M1/70	Biolegend	101226
CD45	BV785	30-F11	Biolegend	103149
CD11c	APC	N418	Biolegend	117310
CD45	BV605	30-F11	Biolegend	103140
CD64	PE-Cy7	X54.5/7.1	Biolegend	139314
MHCII	BV650	M5/114.15.2	Biolegend	107641
CD11b	APC	M1/70	Biolegend	101212
Viability	Zombie UV		Biolegend	423107
Viability	eFluor-450		eBioscience	65-0863-14
